# Rotation flap distraction osteogenesis for unicoronal synostosis

**DOI:** 10.3171/2021.1.FOCVID20124

**Published:** 2021-04-01

**Authors:** Alvin Wong, Arvin R. Wali, Bryan Ryba, Mihir Gupta, Michael L. Levy, Amanda A. Gosman

**Affiliations:** Divisions of 1 Plastic Surgery and; 3 Neurosurgery, Department of Surgery, University of California, San Diego;; 2 Rady Children's Hospital, San Diego; and; 4 University of San Diego School of Medicine, San Diego, California

**Keywords:** unicoronal craniosynostosis, distraction osteogenesis, bone flap

## Abstract

Unicoronal craniosynostosis is notoriously difficult to treat, with long-term studies demonstrating high rates of relapse and the need for reoperation using open fronto-orbital advancement. Applying the principles of distraction osteogenesis to cranial vault remodeling has demonstrated promising short-term results that compare favorably with traditional methods, with simultaneous correction of both frontofacial and endocranial morphology, along with significant increases in intracranial volume. Here, the authors demonstrate their technique for rotation flap distraction osteogenesis in the treatment of unicoronal synostosis and provide case examples.

The video can be found here: https://vimeo.com/519505008.

## Transcript

Unicoronal craniosynostosis is one of the most difficult types of craniosynostosis to treat due to its relatively high incidence of relapse, requiring reoperation in over 20% of patients in some case series. Traditional techniques involving removal of the frontal bone and orbital bandeau, ex situ remodeling, and overcorrection have not successfully avoided relapse of the deformity over time. This was our motivation to develop a technique that would preserve vascularity and growth of the cranial bones and to hopefully achieve more sustainable correction by gradual vault expansion.

**0:50 Introduction**. This video describes our technique for creation of a bony rotational flap for treatment of unicoronal synostosis via distraction osteogenesis.

**0:58 Patient Example**. Here we see an example of a 10-month-old boy presenting with left unicoronal synostosis. He displays the classic hallmarks of the harlequin deformity, including ipsilateral forehead flattening, orbital recession, and nasal root deviation.

**1:11 Patient Example—CT**. His CT scan shows early fusion of the left coronal suture as well as a harlequin deformity of the left orbit.

**1:19 Patient Setup**. Preoperative antibiotics, tranexamic acid, mannitol, and Decadron are given prior to incision. The patient is placed in the supine position on a Mayfield head rest and the bed rotated 180°.

**1:30 Patient Setup**. The scalp is marked with a bicoronal lazy-wave incision. This mark is biased posteriorly on the affected side to compensate for anticipated anterior migration of the incision with distraction. The incision is then infiltrated with 0.25% Marcaine containing epinephrine.

**1:46 Initial Dissection and Temporalis Muscle Dissection**. An incision is made through the scalp and subcutaneous tissues to the level of the subgaleal plane. The scalp is elevated in the subgaleal plane to the level of the frontal orbital bar. A centimeter superior to the supraorbital rims, the periosteum is incised and the dissection continued in a subperiosteal plane over the supraorbital rims and into the orbits bilaterally. On the affected side, the temporalis muscle is then elevated along the anterior third to allow for access to the lateral temporal fossa and sphenoid. The scalp is elevated in the subperiosteal plane along the lateral zygomatic frontal sutures to allow for adequate exposure. The nasal radix was also elevated in a subperiosteal plane.

**2:27 Bone Flap Markings**. We then proceed with marking of the fronto-orbital bar on the affected side with partial extension to the contralateral zygomaticofrontal suture. A partial bifrontal craniotomy was also designed in standard fashion on the affected side. This was extended slightly beyond the midline on the contralateral side and a curved back cut was designed anterior to the intact coronal suture. The apex of rotation is the lateral orbital rim of the contralateral side. The lateral orbital rim acts as the hinge and pedicle of the rotation flap and provides connection to the native vascularized bone and functional sutures.

**3:05 Burr Hole Creation**. Burr holes are then created to allow for insertion of the craniotome. Care is taken to ensure that the burr holes are kept off of the orbital bandeau.

**3:19 Osteotomies**. The dura is elevated off the sphenoid wing and temporal fossa, extending onto the bilateral orbital roofs. An osteotomy is performed along the greater wing of the sphenoid utilizing a reciprocating saw.

**3:35 Ultrasonic Osteotomies**. These osteotomies are then extended into the lateral orbital rim on the affected side with an ultrasonic saw. The orbital osteotomies along the medial wall are connected across the radix. The osteotomy is only partially completed through the unaffected zygomaticofrontal suture.

**3:57 Bone Flap Rotation**. At this point, the frontal craniotomy is allowed to remain in situ, and both it and the fronto-orbital bar are able to be hinged off the unaffected side. The bone flap is not advanced in a straight-line manner, but rather along a curvilinear vector. The osteotomized portion of advancement flap moves anteriorly while the nonosteotomized part stays in situ, giving a rotational movement.

**4:18 Frontal and Bandeau Plating**. The frontal craniotomy flap is then secured to the fronto-orbital bar with resorbable plating, restoring a more convex anatomical relationship between the superior rim and the frontal bone before both segments are rotated forward as a unit.

**4:35 Placement of Distractor**. The distractor appliance is then introduced into the field and situated laterally between the craniotomy flap and the intact posterior vault and secured to allow for an anterior-inferior vector of advancement. While the same overall technique is used in each case, setting distraction vector and amount of overcorrection requires clinical judgment.

**4:53 Distractor Advancement**. The distractor is advanced in situ to ensure the appropriate vector has been chosen. It is fully closed prior to temporalis resuspension and closure.

**5:11 Temporalis Resuspension**. The temporalis muscle is resuspended to the fronto-orbital bar resorbable plates with a back cut to avoid tethering the frontal bone and fronto-orbital bar. The distractor is again activated to ensure that the temporalis was not tethering the advancement and also that there is no buckling along the bony flaps.

**5:40 Closure**. A drain is left beneath the scalp, and the scalp is closed with a series of buried galeal 3-0 Vicryl suture followed by a running 3-0 chromic.

**5:48 Postoperative Care**. Postoperatively, patients are admitted to a regular ward bed. Distraction is started on postoperative day 3 at a rate of 1 mm per day, aiming for overcorrection to compensate for more overall growth on the unaffected side over time. Patients are usually sent home on postoperative day 3. Total distraction averages 27 mm. Following distraction, patients undergo a consolidation period of 8 weeks after which the device is removed in a planned second operation.

**6:12 Postoperative Result**. Three months postoperatively, the patient demonstrates greatly improved forehead and supraorbital rim contour and symmetry and is ready for distractor removal.

**6:25 Postoperative CT**. Postoperative CT scan approximately 3 months postoperatively demonstrates excellent restoration of cranial and facial proportions and osteogenesis occurring in the distracted area.

**6:37 Long-Term Clinical Example—Preoperative. ​**​Here we demonstrate a clinical example of a patient with right unicoronal synostosis who underwent rotational flap distraction osteogenesis.

**6:49 Long-Term Clinical Example—4 Years Postoperative**. Four years following completion of distraction and removal of distractors, the patient demonstrates excellent overall symmetry and durability of repair.

**7:12 References** ^1–5^

## Author Contributions

Primary surgeon: Gosman, Levy. Assistant surgeon: Wong, Wali, Gupta. Editing and drafting the video and abstract: Gosman, Wong, Wali, Ryba, Levy. Critically revising the work: Gosman, Wong, Wali, Gupta, Levy. Reviewed submitted version of the work: Gosman, Wong, Wali, Gupta, Levy. Approved the final version of the work on behalf of all authors: Gosman. Supervision: Gosman, Levy.
